# Quantitative assessment of Ki67 expression in correlation with various breast cancer characteristics and survival rate; cross sectional study

**DOI:** 10.1016/j.amsu.2019.11.005

**Published:** 2019-11-17

**Authors:** Ayad Ahmad Mohammed

**Affiliations:** General Surgery, Department of Surgery, College of Medicine, University of Duhok, Nakhoshkhana Road, 8 AM-1014, DUHOK, Kurdistan Region, Iraq

**Keywords:** Ki67, Breast cancer, Biomarkers, Survival rate, Ductal carcinoma, TNM staging

## Abstract

**Background:**

Ki-67 is a cellular proliferative index that has variable expression during cell cycle. The absence of Ki-67 in the quiescent tissues and its expression in the proliferating cells had linked its possible role in the proliferative capacity of the tissues.

**Materials and methods:**

This retrospective study included 314 patients with breast cancer who underwent various types of breast surgeries. Analyzed was done to find any possible correlation between the level of Ki67 and various patient and tumor characteristics and the survival rates.

**Results:**

The mean age was 48.73 years, the overall survival was 30.77 months, 90.8% of the patients were alive and 8.3% died from breast cancer. In 85.67% no recurrence was reported and 5% had local and axillary recurrences, the commonest sites of metastases were bones and the vertebrae (2.2% and 1.9%) respectively. The mean size of the tumor was 34.39 mm and the mean number of the positive axillary nodes was 4. The expression of Ki67 was around 5–10% in the majority of patients, the mean expression was 28.54%. There was significant correlation between the level of Ki67 and the histopathological grade of the tumor, p value 0.003, and there was no significant correlation with other variables. There was no relation between the overall survival and the Ki67 level.

**Conclusion:**

Ki67 is correlated with the grade of the tumor, and is not a predictor for the survival of breast cancer patients. It may predict aggressive behavior of the tumor and higher histopathological grades.

## Highlights

• Breast cancer is the commonest cause of cancer related deaths among women.
• It has variable biological behavior and prognosis depending on variable factors.
• The usefulness of the Ki67 evaluation as an independent prognostic factor in breast cancer is a matter of great debate.

## 1 Introduction

Breast cancer is the most common cancer affecting females during their life time, it involves a heterogeneous group of tumors that are classified based on various histopathological types, variable genetic bases, and the expression of marker which are determined by immunohistochemical analyses. Breast cancer has variable prognoses depending on many cancer related and patient related factors [[1], [2], [3]].

Although the tumor may have a similar histopathological characteristic, the biological behavior of the tumor varies greatly based on the different molecular expression and hormonal receptor status which depend on genomic variabilities [4].

It has been acknowledged that is a cellular proliferative index, Ki-67 was first identified by Gerdes et al. as a nuclear-protein, shortly after that a corresponding antibody was then described them in the city of Kiel (hence “Ki”) after immunization of mice with the Hodgkin's lymphoma cell line L428 (67 refers to the clone number on the 96-well plate in which it was found). The gene coding for Ki-67 protein is located is on the long arm of human chromosome 10 (10q25) [1,5].

There is variable expression of Ki-67 during cell cycle, its levels are low during the G1-and early S-phase and increased progressively reaching a maximum level during mitosis, then a rapid reduction occur during anaphase and telophase. The half-life of the Ki-67 protein is around 1–1.5 h [1,4].

The absence of Ki-67 in the quiescent tissues and its expression in the proliferating cells had made this marker of great interest to the scientists and linked its possible role in the proliferative capacity of the tissues [1].

The percentage of the positively stained malignant cells for ki67 should be used as expression index or score [6].

Evaluation of the prognostic factors in breast cancer patients is very important step in the management, this will help to direct the possible treatment options and may modify certain chemotherapeutic regimens. When the patient has poor prognostic features more aggressive surgical intervention may be required. Prophylactic mastectomy sometimes is indicated in patients strong genetic and familial predisposition. These prognostic features included the size, the axillary nodal status, the presence or absence or metastatic disease, the grade of the tumor, and the expression of various hormonal receptors and other cell proliferative markers [7,8].

**Research registration:** The research is registered according the World Medical Association's Declaration of Helsinki 2013 at the research registry at the 13th of September 2019, Research registry UIN: research registry 5127.

### 1.1 Materials and methods

This is a retrospective study which included a total number of 314 patients, the study was conducted on patients who had breast cancer and were treated surgically either by modified radical mastectomy or by breast conservation surgery, the operations were done in 3 specialized centers for breast surgery. Data were analyzed regarding the correlation between the level of Ki67 expression and various patient and tumor characteristics and to find any possible relation with the survival rates of the included patients. The specimens were examined for histopathological and immunohistochemistry characteristics; the specimens were fixed in 10% buffered formalin solution embedded in paraffin. Histopathological analyses and the immunohistochemistry interpretation were done by 3 specialist pathologists.

Four μm thickness tissue samples were formed for histopathological assessment of the tumor and immunohistochemical analysis. The assessment was done using standard streptavidin-biotin complex method on automated immunohistochemistry stainer (Dako Autostainer), reagents and buffers were used according to manufacturer guidelines (Dako, Denmark).

Clinical staging of the tumor was done based on the 8th American Joint Committee on Cancer (AJCC) criteria. Histological grade for the tumor was done according to the modified Scarff-Bloom-Richardson Scoring System.

Informed consents were obtained from all the participants to be included in this study. All eligible patients were included, patients in whom the data were not available, who lost from follow up, or those who refused to be included in this study were excluded.

### 1.2 Statistical analysis

The descriptive purposes of our study is displayed in frequency and percentage for categorical variables and mean and standard deviation for continuous variables, different patient categories such as the gender, the age, the site of involvement, the survival status, recurrence, and the overall survival in months were described. The factors related to the tumor were also categorized according to the staging, the histopathological type.

The percentage of the Ki67 expression were described and were correlated to various patients and tumor characteristics using the simple linear regression test.

The level of the Ki67 expression was then separately correlated to the overall survival in months using the scatter/dot graph.

Significant association was determined in P-value of less than 0.05. Data were analyzed using the Statistical Package for Social Sciences (SPSS 24:00 IBM: USA).

The work of this article has been reported in line with the STROCSS criteria [9].

## 2 Results

The mean age of the patients in our study is 48.73 years and females constitutes 99.7%. the overall survival was 30.77 months, 90.8% of the patients were alive during the period of the study, 8.3% died from breast cancer and other died from other causes. In 52.2% the cancer was involving the left breast and in 0.6% there was bilateral involvement. Modified radical mastectomy was performed for 67.2% and the rest underwent breast conservation surgery. Table 1.

**Table 1:** Patients and some tumor characteristics.

Main categories	Subcategories	Frequency	Percent
**Gender**	Females	313	99.7
	Males	1	0.3
**Age in years** (M; SD). Range: 27–83 years.		48.73	11.64
**BMI** (M; SD) Range: 20–45.785		30.72	5.59
**Overall survival in months(M;SD).** Range: 1.45–130.72 months.		30.77	21.16
**Survival status**	Alive	285	90.8
	Died from breast cancer	26	8.3
	Died from non-cancer related causes	1	0.3
	Died from ovarian cancer	1	0.3
	Died from leukemia	1	0.3
**Site involved**	Left breast	164	52.2
	Right breast	148	47.1
	Bilateral	2	0.6
**Type of surgery**	Modified radical mastectomy	211	67.2
	Breast conservation surgery	103	32.8

**Fig. 1:**
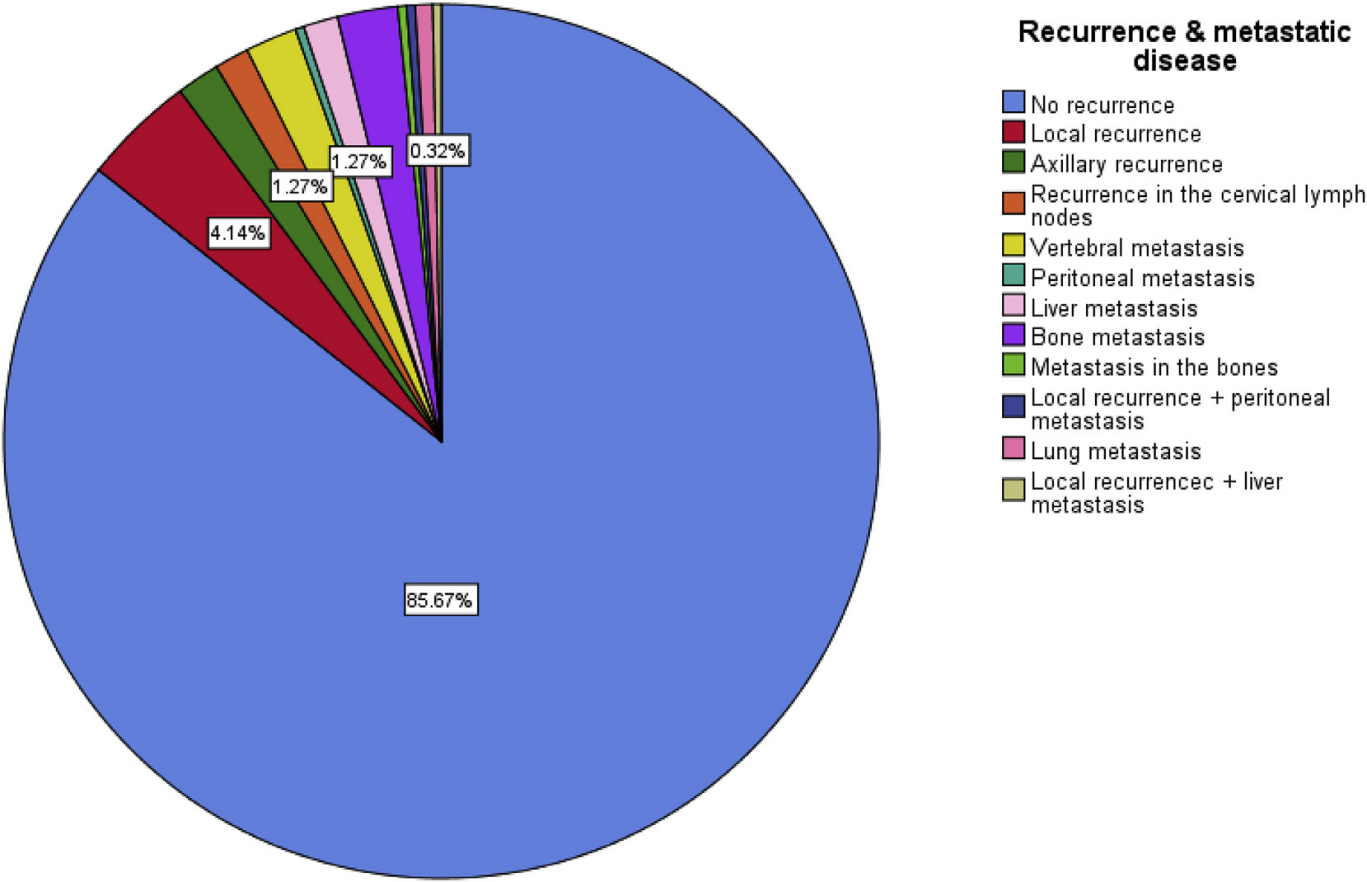
A simple pie chart showing the percentages of the recurrence status and the sites of recurrence.

In 85.67% of patients no recurrence was reported, local and axillary recurrence were reported in around 5% of cases, among the commonest sites of metastatic disease were bone metastasis, vertebral metastasis, cervical lymph nodes, and the liver which were 2.2%, 1.9%, 1.3%, and 1.3% respectively. Fig. 1.

The mean size of the tumor was 34.39 and the number of the positive axillary lymph nodes was 4, stages IIB and IIIA constitutes the commonest clinical stages, 24.8% and 22.6% respectively. Invasive ductal carcinoma/not otherwise specified constitutes the most common histological type. Table 2.

**Table 2:** Tumor characteristic, clinical stages, and histopathological types.

Main category	Subcategories	Frequency	Percentage
Tumor size in mm, Range: 5–100 mm		34.39	16.951
Number of the involved LN, Range: 0–31 LN		4.04	5.462
Tumor grade	Low grade tumor	11	3.5
	Intermediate grade	159	50.6
	High grade	144	45.9
TNM stage	Stage IA	29	9.2
	Stage IB	9	2.9
	Stage IIA	59	18.8
	Stage IIB	78	24.8
	Stage IIIA	71	22.6
	Stage IIIB	12	3.8
	Stage IIIC	35	11.1
	Stage IV	21	6.7
Histopathological type	IDC	23	7.3
	IDC/NOS	247	78.7
	IDC/Comedo type	3	1.0
	IDC + DCIS	5	1.6
	IDC/Medullary type	1	0.3
	ILC	19	6.1
	IDC + Lobular components	1	0.3
	ILC/Pleomorphic type	1	0.3
	IDC/neuroendocrine differentiation	1	0.3
	IBC	4	1.3
	Mucinous carcinoma	3	1.0
	Paget's disease	3	1.0
	Micro-papillary carcinoma	2	0.6

**Fig. 2:**
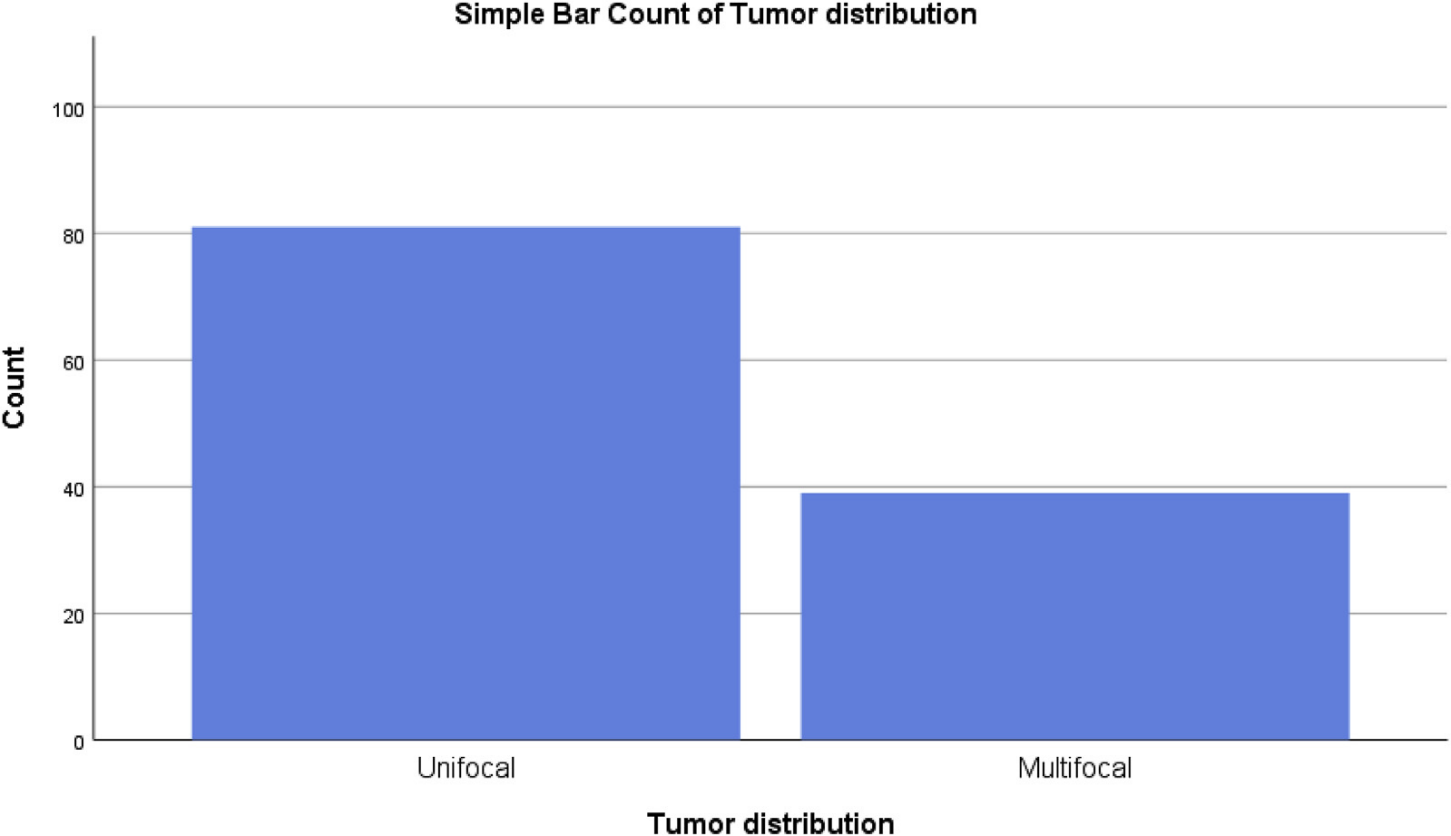
A simple bar chart showing the focality of the tumor.

**Fig. 3:**
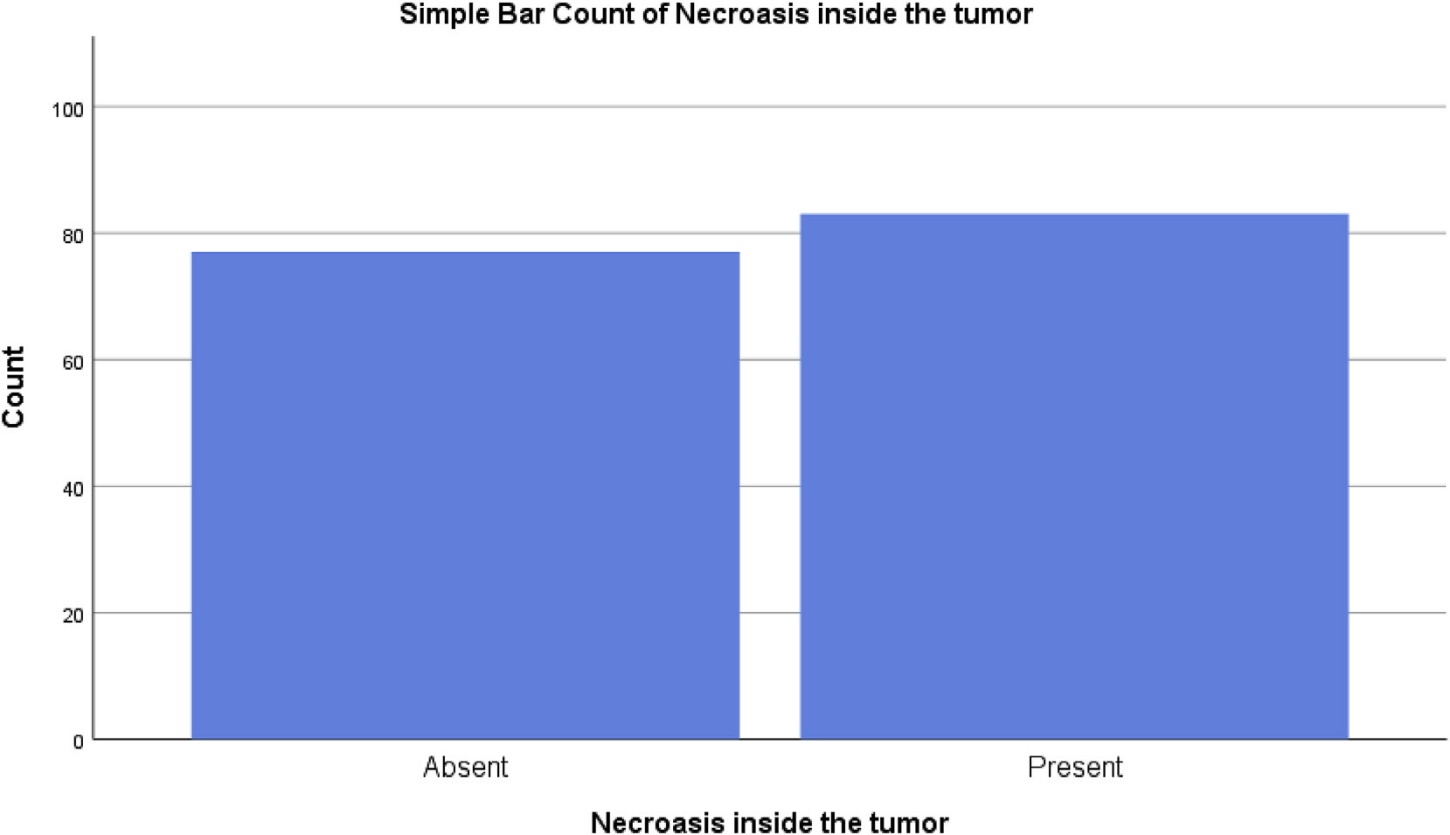
A simple bar chart showing the status of the tumor necrosis.

Most tumors were unifocal and the necrosis was present in more than half of patients. Fig. 2, Fig. 3.

**Fig. 4:**
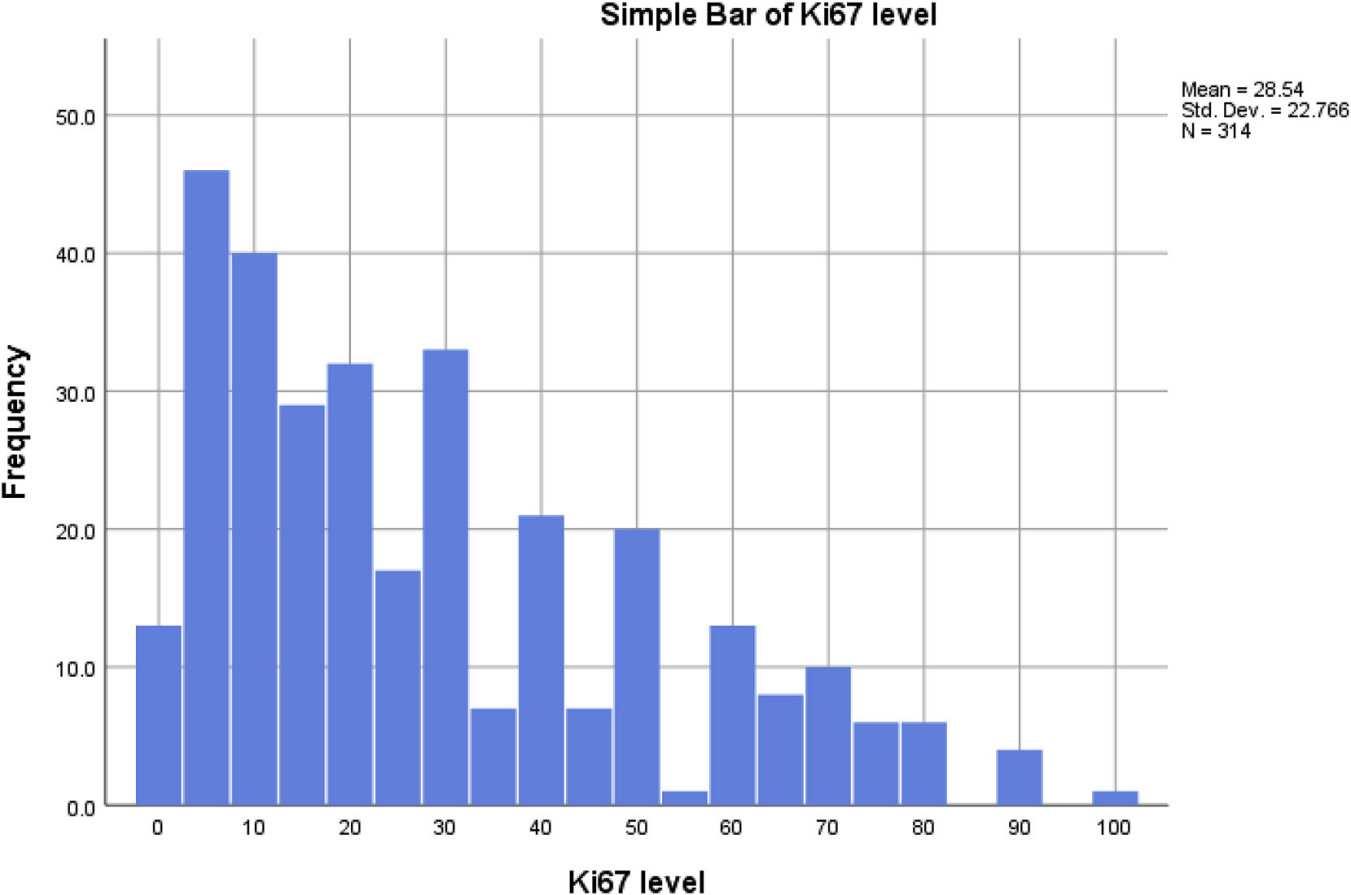
A simple bar chart showing the different levels of KI67 expression in the tumor tissues.

The expression of Ki67 was around 5–10% in the majority of patients, the mean expression of Ki67 was 28.54. Fig. 4.

There was significant correlation between the level of Ki67 and the histopathological grade of the tumor, p value 0.003, and there was no significant correlation with the TNM stage, the survival, the histopathological type, and the other variables. Table 3.

**Table 3:** Showing the correlation between the level of Ki67 and various patient tumor factors using the simple linear regression test.

	Standardized Coefficients	t	Sig.	95.0% Confidence Interval for B	
	Beta			Lower Bound	Upper Bound
Age	−0.108	−0.830	0.412	−0.752	0.315
Site of the tumor	0.077	0.580	0.565	−8.607	15.507
BMI	0.156	1.075	0.289	−0.532	1.734
Survival status	−0.171	−0.707	0.484	−74.931	36.196
Overall survival in months	−0.086	−0.508	0.615	−0.855	0.513
Tumor size (mm)	−0.117	−0.636	0.529	−0.726	0.379
Number of positive LN	0.115	0.806	0.426	−0.747	1.733
TNM stage	0.008	0.054	0.957	−4.218	4.449
Histopathological types	−0.319	−1.889	0.067	−9.139	0.324
Grade of the tumor	0.482	3.126	**0.003**	7.592	35.648
Necrosis inside the tumor	0.039	0.242	0.810	−13.111	16.658
Tumor distribution	0.132	0.886	0.381	−8.281	21.141
Recurrence & metastases	0.205	0.955	0.346	−6.210	17.261

**Fig. 5:**
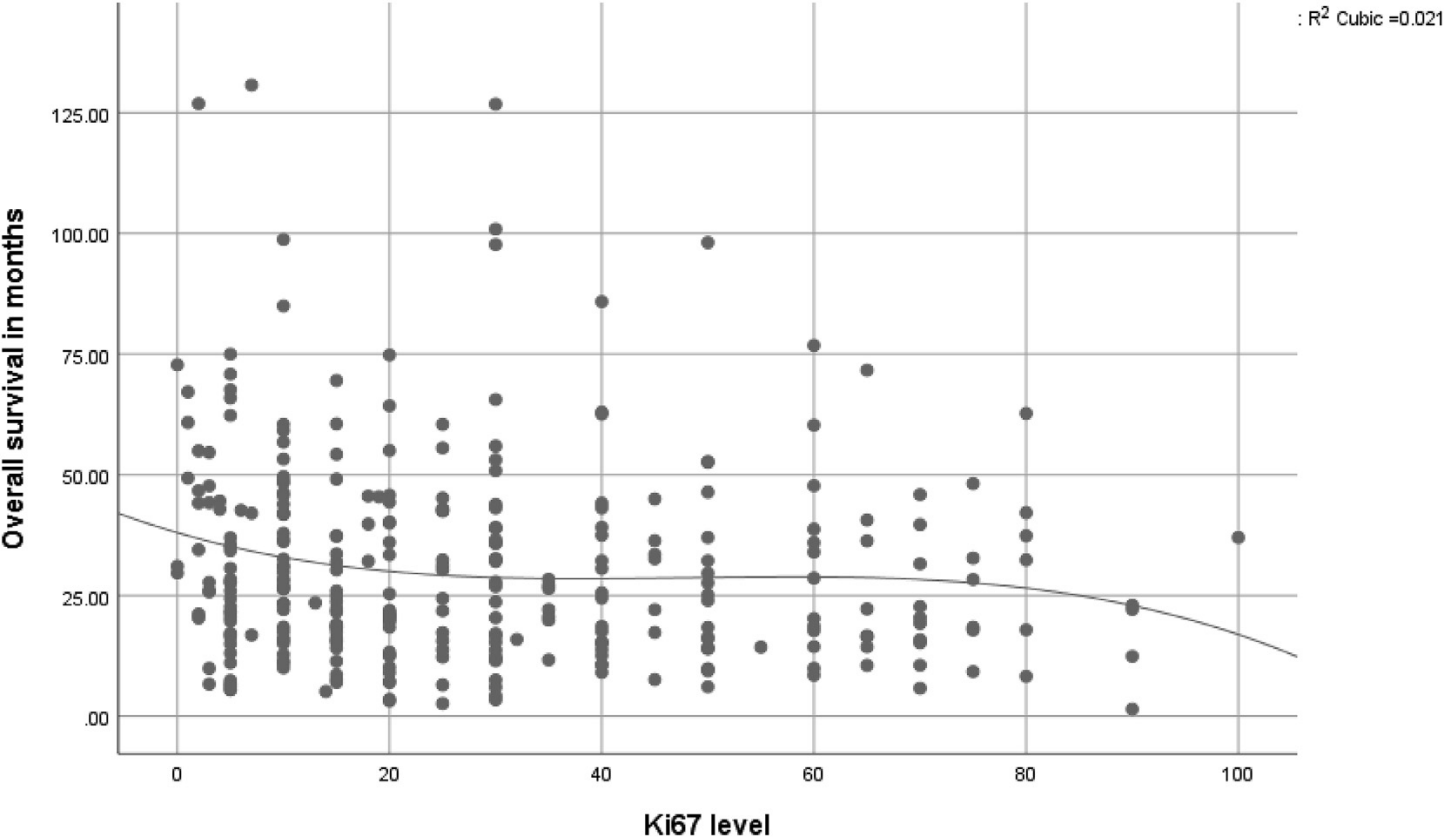
Scatter/dot graph showing the relation between the level of Ki67 and the overall survival in months.

The scatter/dot graph showed no relation between the overall survival and the Ki67 level. Fig. 5.

## 3 Discussion

Many studies evaluated the prognostic significance of Ki67 in breast cancer patients, but the majority of them are retrospective studies and they correlate it with a heterogeneous group of patients. The link between Ki67 and the proliferative activity of breast cancer had been studied since its discovery [6,10].

The usefulness of the Ki67 evaluation as an independent prognostic factor in patients with breast cancer is a matter of great debate between the surgeons, and till now is a matter of open discussion. Many studies have been done to determine various types of correlations between Ki67 levels and various factors in breast cancer and other types of cancers, however larger types of studies based on larger samples are required to prove or exclude this issue [11].

Cell lines varies in terms of expression of Ki67, the higher the expression of Ki67 levels, the poorer is the prognostic outcome of the cancer. Very aggressive cell lines will express a very high level of Ki67 which may reach 100%, in our study the majority of tumor tissues were expressing levels below 50%, and the most common group were ranging between 5 and 30%, only few samples were expressing levels that reaches 90–100% [12].

High levels of ki67 expression may be used to modify the chemotherapy regimen and proved to have some clinical and probably survival benefits in this group of patients. Some authors depend on percentage suppression of Ki67 as an endpoint indicator for residual risk of recurrence after successful treatment [6].

High expression of Ki67 was strongly associated with the higher grades of the tumor in our study, p value 0.003, this result is similar in many studied which showed a similar correlation [3,4].

The majority of our patients underwent modified radical mastectomy (67.2%), and 32.8% underwent breast conservation surgery with sentinel lymph biopsy. There is no prognostic difference between both types of surgeries and the overall survival is the same in most studies [13].

Some authors found a positive correlation between the level of Ki67 expression and the risk of local recurrence, although in our study we didn't find any significant correlation with either the recurrence rate and the distant metastasis, p value 0.955 [3].

There was no statistical association between the Ki67 and the age, the tumor size, and the number of the positive axillary lymph nodes (p values 0.412,0.529, and 0.429) respectively, these results are similar to one of our previous studies in which we correlated the positive and the negative levels of Ki67 and we depended on a cut off level of 14% to categorize the positive and the negative groups because Ki67 is expressed at low levels in normal tissues, however in this study we correlated the quantitative level of Ki67 expression with various patient and tumor characteristics [4,14].

The median age of our patients was 48.73 years (SD; 11.64) which is younger than the results of other studies which included large population of participants. The median survival of the patients enrolled in our study was 30.77 months (SD; 21.16), and the majority were alive at the time of the study (90.8%), 8.3% died from cancer, and the rest died from other causes. There was no correlation between the survival rates and the level of Ki67 expression in our study, p value (0.615), other studies also indicated that the disease free survival is independent on the level of Ki67, many other biomarkers affect the biological behavior of the cancer and the survival rates. In the contrary, some authors found a significant relation between both the overall survival and the disease free survival rates in relation to the cut off levels of Ki67 expression adopting levels less than 12% as negative values [[15], [16], [17], [18]].

The two most common histological types of breast cancer in our patients were invasive ductal carcinoma and invasive lobular carcinoma, the former constitutes more than 85% and the later about 6%, however these percentages are higher compared to other population based studies which showed lower percentages of invasive ductal carcinoma (75%) and higher percentage of invasive lobular carcinoma (15%) among their patients [17].

Most patients had unifocal disease and the intra-tumor necrosis was present in more than half of the patients. More aggressive surgical management is not associated with better outcomes in patients with multicentric and multifocal in terms of loco-regional recurrence, although it can be done when a good cosmetic surgery can be performed [19].

The most common cause of breast cancer related death is uncontrolled metastatic disease, local recurrence is better controlled than metastatic disease, in our study the majority of patients had no clinical and imaging-based evidence of recurrence or metastasis (85.67%), around 5% had local or axillary recurrence. The most common site for metastatic disease was the bone which was reported in 2.2% of our patients, the bones affected were the femur, the humerus, the pelvic bones and the scapula. Vertebral metastasis was the next most common site which was reported in 1.9% of our patients, liver and cervical lymph nodes metastasis was reported in 1.3% for each group. In most studies the bones are the commonest sites of metastatic breast cancer followed by the lungs then the liver and these results are close to our findings, however in our results we evaluated the bone and the vertebral and other bone metastasis separately [5].

There is no evidence based on the clinical protocols for the routine use in clinical practice, but the pathologist must follow some standardized guidelines for the assessment multidisciplinary team [20].

Guidelines include a high Ki67 level as an indicator for increased risk of recurrence in patients who have estrogen receptor positive, HER-2-receptor negative bract cancer patients, this may indirectly support the need for the modification of the endocrine and the chemotherapy regimen in such patients [10].

Our data indicate that the labeling index of Ki67 expression is associated with higher tumor grades and doesn't indicate a strong indicator of other features of poor prognostic outcome.

## Provenance and peer review

Not commissioned externally peer reviewed.

## Ethical Approval

NA.

## Sources of funding

No source of funding other than the authors.

## Author contribution

Study design, data collection and analysis, writing and final approval of the manuscript: Dr Ayad Ahmad Mohammed.

## Trial registry number

N/A.

## Guarantor

Dr Ayad Ahmad Mohammed.

## Declaration of competing interest

No conflicts of interest present.
